# Context as Relevance-Driven Abduction and Charitable Satisficing

**DOI:** 10.3389/fpsyg.2016.00305

**Published:** 2016-03-08

**Authors:** Salvatore Attardo

**Affiliations:** College of Humanities, Social Sciences, and Arts, Texas A&M University-CommerceCommerce, TX, USA

**Keywords:** context, linguistics, pragmatics, cooperative principle, principle of charity, relevance, abduction, satisficing

## Abstract

It has been widely assumed that the full meaning of a linguistic expression can be grasped only within a situation, the context of the utterance. There is even agreement that certain factors within the situation are particularly significant, including gestures and facial expressions of the participants, their social roles, the setting of the exchange, the objects surrounding the participants, the linguistic, cultural and educational backgrounds of the participants, their beliefs, including those concerning the situation, the social procedures and conventions that regulate the situation. Finally, there is some agreement that context is dynamic, reflexive (the speakers are mutually aware of their beliefs), not limited to linguistics actions, and last but not least, a psychological construct. This definition of context is not (very) controversial, but it leaves out two major problems, which will be addressed in this paper: how is context arrived at? And, since a perfectly natural interpretation of the above definition could be that the context of each utterance is the entire universe, how is the relevant context delimited? Four related concepts will provide the answer to both questions: abductive reasoning, driven by relevance and cooperation, and bounded rationality and the principle of charity. Simply put, context is derived abductively by the speakers assuming that for the speakers to behave the way they behave and do so rationally, a given context must be available to them. The context is bounded by the simple requirement that speakers not try to optimize their interpretation/calculation, but rather satisfice, i.e., find the first acceptable solution and by the need to follow the principle of charity, which forces intersubjective agreement. Thus, abductive reasoning and bounded rationality will be shown to be sufficient to calculate the relevant context of utterances (or other rationality-driven interactions) and to effectively delimit the potentially infinite search space that must be explored to do so.

I would like to begin discussing context by using a metaphor^1^. As is well known, metaphors have heuristic powers, which will help us in this complex and fraught subject. The metaphor is that studying context is akin to studying non-foveal vision. Peripheral (non-foveal) vision is quite important in many situations (for example, in driving one becomes aware of the presence of a car passing to the left or right through non-foveal vision, at first). This image helps us realize that context exists only in opposition to a text. A context never exists by itself. It exists because it is something other than the text. However, the metaphor is even more interesting, because it highlights another feature of context: if we notice something in our non-foveal vision and we shift of visual angle to focus on the thing (for example, by turning one's head to look at the car passing us on the right lane) then the object is no longer part of the non-foveal vision, but it is now in the foveal vision angle. To put it differently, one cannot study peripheral vision by focusing on it, because the act of focusing on it changes radically the nature of the thing to be observed.

As we will see in the present paper, most of the history of the research on context has consisted, roughly speaking, of focusing our gaze in the general direction of the object glanced at in peripheral vision and then trying to describe or enumerate the salient features of what is seen. I will argue that that approach misses largely, but not completely, the point. The remainder of the paper will be organized in two main parts: the first one will provide a cursory and non-representative, but nonetheless enlightening review of definitions of context, primarily within linguistics, but with some extracurricular forays. The second part will present the constructive side of the paper, presenting some of the tools needed to derive and bound context. I should stress that the present discussion should not be read as antagonistic to but rather as complementary to traditional definitions of context.

## A partial history of context

There are as many approaches to context as there exist disciplines in the humanities and the social sciences. It would be unrealistic to attempt to encompass them all. Therefore, I have settled for presenting a largely linguistic overview of definitions of context, to the detriment of other disciplines, such as psychology, philosophy, and phenomenology.

### Context free grammars

We will begin this review of definition of context, perhaps perversely, with the zero-degree of the term, or more specifically with Bloomfield rejection of the tractability of the very idea of meaning, let alone context. Bloomfield, famously rejected mentalistic psychology and espoused behaviorism. For Bloomfield, since the meaning/context continuum is potentially infinite it is *eo ipso* intractable:

We have defined the meaning of a linguistic form as the situation in which the speaker utters it and the response which it calls forth in the hearer.…In order to give a scientifically accurate definition of meaning for every form of a language, *we should have to have a scientifically accurate knowledge of everything in the speakers' world*. The actual extent of human knowledge is very small compared to this.…The statement of meanings is therefore the weak point in language-study, and will remain so until human knowledge advances very far beyond its present state (Bloomfield, 1933, pp. 139–140; my italics, SA).

Bloomfield believed that sub-morphemic analysis of meaning was impossible: “There is nothing in the structure of morphemes like wolf, fox, and dog to tell us the relation between their meanings; this is a problem for the zoölogist.” (1933: p. 162). However, as Langendoen (1998) points out, by the time the American Structuralist school had reached its peak, sub-morphemic analyses of semantics were being performed (e.g., Goodenough, 1956). The view that the semantic content of a morpheme can be broken down in semantic features was incorporated in Katz and Fodor's (1963) semantics, which became the de facto semantics of generative grammar (Chomsky, 1965). As the name itself of the type of grammar strongly suggest, context-free grammars were not sensitive to, or interested in, context. The idea behind context-free grammars is that rewriting rules and transformations did their work regardless of the context in which they occurred. NP rewrites as Art + N, regardless if the NP is the first or the last of a sentence.

Generative semantics attacked the context-free nature of generative grammar “semantics” (the scare quotes acknowledge the reluctance that many generative grammarians would have had in using the term) using a barrage of examples such as the following:

(1) “John called Mary a republican and then *she* insulted *him*.” (Lakoff, 1971: p. 333)

Example (1) above, in the emphatic prosody reading, works only if we assume that Mary considers “Republican” an insult. Or to put it differently, we need to know what Mary's state of mind is, in order to decide on the intonation of the sentence. Clearly, someone's state of mind cannot be part of the morphemic meaning of a sentence

Worse, even such a concept as grammaticality, one of the core ideas of generative linguistics, could be show to depend heavily on context, with examples such as the following:

(2) “Kissinger conjectures poached.” (McCawley, 1976/1979)

Example (2) would be rejected by most speakers of English as non-grammatical, unless they can imagine it as the answer to the question: “How does President Nixon like his eggs in the morning?”

As is well known, generative semantics self-destroyed (Harris, 1993) and was reborn as pragmatics. We take up that thread next.

### Pragmatics

The linguistic tradition that Bloomfield was reacting against in 1933 came from such German thinkers as Humboldt and Wegener. Humboldt makes it very clear that, for him, meaning does not come just from the forms of language but from the “act of speaking” (see Nerlich and Clarke, 1996: p. 53). Wegener claims that interpretation depends on the “situation” Wegener (1885) and named his entire theory “*situationstheorie*.” Wegener contemplates three types of “situations”: the objective observations (views), the elements associated with the situation by memory, and the (self-) awareness of the participants.

- die Situation der Anschauung [view] (1985: p. 21)
- die Situation der Erinnerung [memory] (1985: p. 22)
- die Situation des Bewusstseins [awareness] (1885: pp. 22–23).

Other factors in Wegener's definition are the “ongoing or just completed activity” and the *Kultursituation* [historical culture] (Knobloch, 1991: p. XVI). Wegener also anticipates speech act theory and Gricean pragmatics, witness the following quote:

Wegener was among the first to realize that speaking and understanding are preconditioned by and embedded in practical action and also dependent on the cooperation among the speakers (Knobloch, 1991: p. XVI).

The German tradition of seeing linguistic meaning as part of a broader context, found a fertile ground in the work of Malinowski. Malinowski is considered the founder of the pragmatic concept of context: he is considered the “first to use [the term] context in a systematic way” Nerlich and Clarke (1996: p. 316). The following quote makes the central role of the pragmatic context in Malinoski's thought very clear:

language […] has an essential pragmatic character […] it is a mode of behavior, an indispensable element of concerted human action (Malinowski, 1923: p. 316)

whereas the following is a definition of context, from Malinowski's masterpiece, *Coral Gardens and Their Magic*:

it is very profitable in linguistics to widen the concept of context so that it embraces not only spoken words but facial expression, gesture, bodily activities, the whole group of people present during an exchange of utterances and the part of the environment on which these people are engaged (Malinowski, 1935 vol. II: p. 22)

Malinowski's influence, concerning the concept of context, on the London School cannot be exaggerated. Raymond Firth, in a 1956 piece on Malinowski, states that Malinowski “has been one of the outstanding influences in shaping modern British social anthropology” (Firth R., 1956: p. 1). Both J. R. Firth and Halliday will be influenced by Malinowski's definition, but also Hymes' (1972) definition of ethnography of speaking is strongly reminiscent of Malinowski's definition of context. Senft (2007) goes so far as claiming that

the factors Hymes (1972: p. 65) summarizes in his famous acronym SPEAKING—“settings, participants, ends, act sequences, keys, instrumentalities, norms,” and “genres”—are not only constitutive for the ‘ethnography of speaking’paradigm but also for Malinowski's “context of situation” (Senft, 2007: p. 148)

J. R. Firth acknowledges Wegener's and Malinowski's influence very directly: “The key concept of the semantic theory he [Malinowski] found most useful for his work on native languages was the notion of context of situation” (Firth J. R., 1956: p. 101).

Firth's own definition of “context of situation”: is as follows:

the linguistic text (…) finds a place and function in relation to other categories such as the participants, relevant non-verbal behavior, relevant objects and effect or result (Firth, 1957: p. 7)

Within the London school, which comprises Malinowski and Firth, the author who has had the broadest impact on linguistics is probably Halliday, who, somewhat ironically, emigrated to Australia in the mid 1970es. Halliday's definition of context is formulated in terms of cultural meanings, but is also influenced by Firth, for example in the insistence that speech is an act of meaning:

Context is (…) a construct of cultural meanings, realized functionally in the form of acts of meaning in the various semiotic modes, of which language is one. The ongoing processes of linguistic choice, whereby a speaker is selecting within the resources of the linguistic system, are effectively cultural choices, and acts of meaning are cultural acts (Halliday, 1971: p. 165).

Context, in Halliday's functional model is articulated in terms of field, tenor, and mode, but a discussion of his model would take us too far afield. In a different context, namely a discussion of intellegibility in spoken language, and 20 years earlier, Catford also defines context in ther now familiar terms of the speakers, the situation, and culture. His definition can be summed up as:

- Speaker and Hearer
- Relative positions and actions at the moment of utterance
- Various objects in the surroundings and their relations to S and H
- H's linguistic background and experiences as well as educational and cultural background (Catford, 1950)

With Ochs (1979) definition of context, we are fully in the domain of interactional sociolinguistics. Accordingly, the following feature prominently in her definition of context:

- The immediate physical environment
- The verbal environment
- The social and psychological world in which the language user operates at any given time
- The above is filtered by the world view of the speakers
- The behavioral environment, defined as “the […] cultural filtering that […] turns physical behavior into conventional acts and events” (1979: p. 2) e.g., “the procedures for entering into and sustaining a state of mutual involvement” (1979: p. 3) such as gaze matching (eye contact)
- The extra-situational context: the speakers' beliefs and understanding of the situation.

However, despite her socio-interactional orientation, Ochs' definition reflects the zeitgeist of when it was presented, being still very tied to the linguistic form. For example, Ochs discusses extensively the grammaticalization of context.

Ochs' definition proved very influential. Duranti and Goodwin (1992: p. 6) explicitly take Ochs' (1979) definition as their starting point. The parameters they use are as follows:

- Setting: social and spatial framework
- Behavioral environment: “the way that participants use their bodies and behavior as a resource for framing and organizing their talk” (1992: p. 7)
- Language (co-text; contextualization cues; genres)
- Extrasituational context: background knowledge

Their discussion ranges very widely, to reach the conclusion that context should not be seen as “a set of variables [the parameters listed above] that statically surround strips of talk” (1992: p. 31) but rather as having a “mutually reflexive relationship to each other, with talk, and the interpretive work it generates, shaping context as much as context shapes talk.” (Ibid.) The other major contribution that Duranti and Goodwin provide is a focus on the relationship between talk and context, which they see as paralleling that between figure and ground. The figure would here be talk (the text) and the ground would be the context. This is a crucial aspect of the definition of context, which Fetzer (2004: p. 3) calls the “core meaning” found, according to her, in “all the usages” of the term “context.”

Despite approaching context from the perspective of the agents, recognizing the figure/ground relationship between text and context, and acknowledging that language is a part of a “stream of activity” (1992: p. 3), Duranti and Goodwin's definition is still very language-centric.

Jakob Mey, long the editor of the Journal of Pragmatics, presented in his pragmatics textbook a definition of context, slightly clarified in the second edition of the book, which follows below:

Context is a dynamic, not a static concept: it is to be understood as the continually changing surroundings, in the widest sense, that enable the participants in the communication process to interact, and in which the linguistic expressions of their interaction become intelligible (Mey, 2001: p. 39)

This definition has the great merit of stressing the dynamic nature of context, which of courses entails the fact that it is enacted and brought into being by the actors in the situation and not somehow per-existing them. The other aspect that should be stressed is that language performance becomes intelligible only within the context, again not pre-existing it.

Finally, some aspects of Schegloff's (1992) discussion of context fit in with the approach presented in this paper. Schegloff recognizes the potential infinity of contexts (see below) and upholds a dynamic view of context: the text helps determine (“invokes”) the context: “the [text] (…) may be understood as displaying which out of that potential infinity of contexts (…) should be treated as relevant and consequential” (Schegloff, 1992: p. 197)^2^.

### Philosophy and psychology

Philosophers have developed highly technical notions of context that need not detain us. However, it is worth considering Stalnaker's “informal (…) intuitive” definition of context:

[T]he concrete situation in which a conversation takes place, a situation with a more or less definite group of participants with certain beliefs, including beliefs about what the others know and believe, and certain interests and purposes, and interests and purposes that are recognized to diverge. (…) intelligible independently of any institutional linguistic practice (…) not defined by the constitutive rules of some language game (…). It is not just linguistic actions, but actions of any kind that take place in a context (Stalnaker, 2014: p. 14)

Let us note the very significant “divorce” of the concept of context from linguistic behavior (“not just linguistic actions”) and the iterative nature of the common ground (“beliefs about what the others know and believe”), familiar from Gricean semantics. Stalnaker also notes that context is a dynamic concept which evolves “in the course of a conversational exchange” (2014: p. 14).

There are numerous definitions of context in psychology, but I will not be concerned with them. Instead, I will consider a psychological definition of context advanced within Relevance Theory:

context is a *psychological construct*, a subset of the hearer's assumptions about the world. It is these assumptions, of course, rather than the actual state of the world, that affect the interpretation of an utterance. A context in this sense is not limited to information about the immediate physical environment or the immediately preceding utterances: expectations about the future, scientific hypotheses or religious beliefs, anecdotal memories, general cultural assumptions, beliefs about the mental state of the speaker, may all play a role in interpretation. (Sperber and Wilson, 1986: pp. 15–16; my emphasis, SA)

What is relevant in Sperber and Wilson's definition is not the typically expansive listing of factors and components of the context, but the clear and important realization that the context of an utterance is not a physical reality but the mental representations of the physical reality, or as they put it that context is a psychological construct. Context does not exist “out there” in the world. It exists “in the head” of the speakers/interactants.

The risk with this definition is to attempt to make it fit the standard “toolkit” of propositional logic, as for example the following claim that an utterance conveys

a *propositional* complex that contains both explicit and implicit information. (…) this information is constructed on the fly as the interpreter processes every lexical item (…) While (…) the propositional complex communicated by an utterance is pragmatically narrowed and simultaneously pragmatically broadened in order to incorporate only the set of *optimally* relevant propositions (…) (Assimakopoulos, 2006: p. 1; emphasis mine, SA)

Unless we assume that all information is propositional by definition, which would render the word “propositional” hard to define, there is no guarantee that all implicatures are propositional. Consider the difference between:

- Dinner is ready.
  and
- The chow is ready.
  which is certainly not propositional, as presumably both would be represented as
- ready(x)

whereas the connotations evoked, even outside of a rich context, are clearly different. We will address the assumption that optimal relevance needs to be sought below.

Finally, we can conclude this selective review of the literature with a book that defies classification, but that, given the title (*Context as other minds*), I chose to list among the psychological approaches. Givón (2005) is indeed such a wide-ranging discussion that I will merely mention a few “recurrent themes” (the title of a section of the first introductory chapter) that are clearly related to the idea of context, to a greater or lesser extent.

We can start with the idea of relevance, which is key to the expansion of the search of the contex, starting from the text. Abduction and analogical reasoning figure prominently on Givón's list. As we will see, relevance and abduction are central to the discussion below. Givón also lists the concepts of similarity, analogy, and metaphor which all are “dependent on the choice of relevant context” (2005; p. 9) and other concepts such as categorization and taxonomy which are dependent on “the capacity to tell “major” traits” (2005: p. 9). While the connections between all these concepts and the idea of context may be not immediately obvious, it is clear that similarity, analogy and metaphor “work” in relation to a frame (a context): an argument is like a war, but only up to a point: if one of the participants starts raising an army, or bombing some territory we are no longer facing an argument. A whale and a submarine are similar, in the for example they both function under water, but obviously also very different. Along the same lines, dogs and coyotes are similar, but of course also different, as captured by their Linnaen taxonomy. Likewise, it would be absurd to argue that Fluffy, one's beloved poodle, is a completely different dog than the neighbor's mongrel, and should belong to a different genus.

Before leaving off the psychological and pragmatic theories, we can address a point suggested to me by one of the referees, who asks to compare the current approach to “social” theories such as common ground (e.g., Clark, 1996) and the “theory of mind” (ToM; e.g., Wimmer and Perner, 1983). Both common ground and ToM “complicate” the notion of context by making it relative to two or more participants, in the sense that the parties must “share” some amount of knowledge. Obviously, context is shared, to an extent, by the various parties that are co-participants in the situation. To be aware of which parts of context are shared and which parts are only available to an individual (or the self) is a crucial need for the participants. However, the present approach applies to the construction of context regardless of whether it is shared or not: the expansive and delimitative principles need to apply regardless of the shared nature of it. Imagine, as Gedankenexperiment, a solitary climber on the face of a mountain. In a difficult spot of the climb, she says: “You can do it!” Assume, for the sake of the argument, that we treat this is speech directed to herself, and not as an imaginary conversation between the climber's self and another self. The principles that would govern a shared interpretation of “do” (namely, climb this mountain) obviously must also govern a solipsistic interpretation.

### Literature review conclusions

There are a few recurring, central ideas that have emerged and that are, in my mind, crucial to the understanding of context. I will review them briefly and then move on to the proactive part of the paper. The first idea is that context is not immanent, it does not pre-exist the communicative exchange and/or the speakers' consciousness. The second idea is that context is a mental state that is constructed by the speakers and/or participants to the situation “on the fly” as they go about their business in the conversation/interaction. Specifically, context is constructed along the lines of relevance and abductively, hence it is largely a matter of implicatures. Context is bounded, i.e., it is not infinite. Finally, we may note two competing forces at play: one is an expansionist force that impels speakers or interactants to seek out relevant parts of the environment to make sense of the text/events. The other, less visible, but just as important, that bounds the expansive search for relevant context, so that it is limited effectively to what is necessary for the purposes of the speakers/interactants. What follows examines the two tendencies.

## Deriving and bounding context

I believe that one observation that emerges from the consideration of the various definitions of context is that there is an expansive tendency: the definitions get more and more complicated in an effort to encompass all possible relevant contextual factors. While that is understandable and even probably necessary, it creates a problem, to which we turn next.

Fetzer (2004: p. 3) notes that “context can refer to the whole universe.” We might add, just to ante up a little, that within the multiverse cosmological theory context might refer to numerous, in fact infinite, universes. As Fetzer concludes, “that extremely general definition of context requires some delimitation” (2004: p. 3) if for not other reason that the computability and psychological reality of an infinite set of concepts is questionable.

The problem, of course, is that even if many have proposed various theoretical constructs to define the domain of context, some of which are reviewed by Fetzer, very few if any scholars have explained how practically context is delimited by the participants of the interaction. Ex post facto, it is always possible to look at a conversation and find that socio-economical factors were at play in delimiting the context to members of a given socio-economical group (for example, the expression “he lived alone with his servants^3^” presupposes that the servants do not count as people, since “alone” requires the absence of others). However, it is unlikely that the writer of the sentence above was aware of and deliberately wanted to express this fact^4^. So, if the delimitation of context is subconscious, happens in real time, and is intersubjective (i.e., the participants to the conversation share it or agree to it, implicitly), how can the speakers take care of it? Moreover, it should not escape our attention that the derivation of context takes place in real time (i.e., as the interaction takes place or the conversation unfolds) since the speakers and the context, as Duranti and Goodwin remind us, are in a dialectical relationship.

### Deriving context: relevance, cooperation, abduction

It is clear that relevance is the tool used to expand the context: the search for relevant implicatures or other implicit parts of meaning obviously drives the process whereby speakers look for features in the situation that may be relevant to what was said. For example, if speaker A says “hold this” while handing a hammer to speaker B, relevance determines that the reference of “this” is the hammer. A's order/request could be paraphrased as a request to hold the item identified by a deictic and the gesture in the situation resolves the ambiguity, under the assumption of relevance. If speaker A had wanted B to hold a book, why hand them a hammer?

Along the same lines, it is obvious that an assumption of cooperation is necessary to process the search for the relevant context: unless he/she assumes that speaker A means what he/she said, is being clear about it, etc. speaker B would have no reason to assume that A wants him/her to hold the hammer and isn't going to drop the hammer or is trying to sell them the hammer, or is a lunatic who likes holding hammers in their hand.

I assume that the reader is familiar with the Principle of Cooperation, proposed by Paul Grice (see Grice, 1989). Likewise, I assume that relevance is a known pragmatic principle or maxim (see Sperber and Wilson, 1986). This is not the place to review the discussion on the subjects, so I will not discuss them further. Instead I will briefly deal with adbuction, which is definitely less known in linguistic circles. A fuller discussion can be found in Attardo (2003).

Abduction, “discovered” by Peirce (1960-1966), is a “third” form of reasoning, besides induction and deduction. The general form of abduction is as follows:

The surprising fact, C, is observed; But if A were true, C would be a matter of course, Hence, there is reason to suspect that A is true (Peirce 5 p. 189).

Clearly abduction is not a matter of certainty: it is a probabilistic “guess” to the best explanation. Moreover, the formulation of the rule (A) which explains C is not itself part of the abductive process for Peirce and so has to be justified externally. Some scholars (e.g., Hoffmann, 1999: pp. 281–284) argue that the generation of A is itself abductive, which open the possibility of a regression ad infinitum.

Other strategies are possible. Brogaard (1999: p. 141) stresses the role of “unexpected or sudden regularities” (Peirce has “surprising”) in triggering the abductive process. Regularities are perceived against the background of observed facts (Kapitan, 1997: p. 482) that are “separated from other facts” (Kapitan, 1997), in fact, “[a]n essential step in the process of abduction is the classification whereby a particular assembly of phenomena comes to be regarded as a single explanandum.” (Kapitan, 1997) So, according to Brogaard (1999), the process of abduction works as follows: the subject observes an undifferentiated stream of phenomena, at some point in time, some of these phenomena exhibit some common feature, which leads the subject to group the phenomena in a single explanandum, furthermore, the presence of unexpected or surprising regularity in the phenomena leads the subject to the formation of an hypothesis which explains the phenomena, if true. The subject then assumes prima facie the truth of the hypothesis. Presumably the explanandum is more abstract than the mere collection of phenomena, which strikes me as a significant part of the explanatory power of the abductive hypothesis.

Other strategies have been used to ground the abductive process, but I believe the present discussion is sufficient to show how the search for a satisfactory context to explain the speakers' utterances will be largely abductive in nature. The metaphor of non-foveal vision I used at the beginning of the paper comes in handy now as well. Much like non-foveal vision cannot rely on foveal fixation, context searching cannot rely only on what is inferrable or deductible from what is literally said in the utterance or on what is said and its pragmatic enrichments. Context searching and building needs to rely on adbuctive jumps. If my wife walks into my office and asks “are you hungry?” to assume that the time of the day (around noon) is relevant and that therefore she is asking me to prepare lunch can only be an abductive process. No logical inference ever could bridge the gap between a question about my inner states and a request to prepare a meal. Note that the time of the day, our habits (I feed the humans, she feeds the animals), what we are doing (both of us are working on papers), and numerous other factors contribute to the successful abduction. These could never be accounted for in inferential or deductive reasoning, as a matter of principle as the list is open ended.

### Bounding context: satisficing and charity

The speakers are in need of a context-delimiting algorithm. I propose the following as a first approximation.

1. Start from the immediate physical environment in which the utterance is produced.
2. Use relevance-driven abductive implicatures to expand the context as needed.
3. Stop when you reach the smallest construct consistent with the principle of charity.
  - To put it differently, stop when you can make sense of what the speakers are saying and/or doing.
  - To put it yet differently, satisfice to when the speakers/agents make sense.

#### Bounding through satisficing

Simon (1983) proposed the idea of bounded rationality (reasoning), i.e., a much more “realistic” view of rationality based on limited knowledge and limited resources, which does not arrive at optimal solutions. Simon introduced in the definition of a (bounded) rational agent the following features:

1. the agent does not optimize (this is called “satisficing” [satisfy + suffice])
2. the agent does not guarantee consistency

The significance of these decisions is great: there is no guarantee that the agent will find an “optimal” (best) solution, because satisficing will lead to accepting a solution that achieves a given goal, when a better option might have been available. Similarly, because not all possibilities are searched, inconsistent facts may be present in the system. Inconsistency and suboptimality are problems, but a conception of reasoning that is bounded is preferable to conceptions of optimal rationality because, simply put, real agents in the real world never have perfect information and therefore bounded reasoning is more realistic. As a side note, we can observe that bounded reasoning solves the problems associated with the need to find optimal relevance, in some formulations of relevance, since bounded searches for a solution are guaranteed to find a solution, although it may not be the best one.

#### Charity

We now turn to a discussion of the principle of charity, which is of necessity longer and more complex than the discussion of all the other tools we have examined so far, for the connected reasons that, within linguistics, virtually no use has been made of the principle of charity and, within philosophy, it has been applied to different problems than those to which we have applied it here.

There have been several proposals of charity principles. In the specific sense that we are interested in, we may begin with Wilson's (1959: p. 532) “principle of charity” which states that “We select as designatum [the referent of a proper noun] that individual which will make the largest possible number of (…) statements true.” Wilson's discussion is technical and need not detain us further, but the basic idea is clear: interpret speech so as to maximize the truth of what the speaker says.

Quine (1960) generalizes the principle of charity, in the context of his discussion of the feasibility of radical translation (i.e., between non-related languages which have never been in contact; Quine, 1960: p. 28). Quine essentially says that if a speaker says something that seems clearly false, a bad translation is more likely than imputing irrationality to the speaker.

Grandy introduces a “humanity principle” in the same context. Grandy is critical of Quine's definition and replaces it with the assumption that “the purpose of translation is to enable the translator to make the best possible predictions and to offer the best possible explanations of the translate” (Grandy, 1973: p. 442). Since obtaining a complete account of the translatee's beliefs and desires is practically impossible, (Grandy argues that all or at least many psychological states can be reduced to these) Grandy concludes that

we use ourselves in order to arrive at the prediction: we consider what we should do if we had the relevant beliefs and desires. Whether our simulation of the other person is successful will depend heavily on the similarity of his belief-and-desire network to our own.(…) it is of fundamental importance to make the interrelations between these attitudes as similar as possible to our own. If a translation tells us that the other person's beliefs and desires are connected in a way that is too bizarre for us to make sense of, then the translation is useless for our purposes. So we have, as a pragmatic concern on translation, the condition that the imputed pattern of relations among beliefs, desires and the world be as similar to our own as possible. This principle I shall call the principle of humanity (Grandy, 1973: p. 443).

The most significant discussion of a principle of charity is to be found in Davidson's philosophy. There is not a single, standard discussion of Charity in Davidson's work; rather, his observations on the Principle of Charity are scattered throughout his work. We can start with one of the last presentations of the principle:

the principle directs the interpreter to translate or interpret so as to read some of his[/her] own standards of truth into the pattern of sentences held true by the speaker. The point of the principle is to *make the speaker intelligible*, since too great deviations from consistency and correctness leave no common ground on which to judge either conformity or difference (2001: p. 148; my emphasis, SA)

The emphasized passage clearly shows that, in Davidson's model, without an assumption of a charitable reading, communication would be impossible. Davidson insists repeatedly that his conception of charity is both indebted to Quine (and to Wilson, via Quine) and significantly different from his, since Quine applies it only to logical operators, whereas Davidson insists repeatedly that his Charity applies “across the board,” i.e., is a general interpretive principle (1984: p. xvii; 1984: p. 153; 2001: p. 148). As Jackman (2003) notes, Davidson's charity principle is broader than Wilson's or Quine's, since it is supposed to determine not only referential semantic issues, but also which propositions are (likely) to be true (or at least believed to be so, by the speaker).

Charity, in Davidson's work, is a central tenet because it allows him to bridge between observable external behavior of the participants and their beliefs/desires. The importance of this step is of course due to his adherence to the behaviorist tenets that only observable behavior could be relied on in scientific work. Needless to say, behaviorism was discredited by modern linguistics (Chomsky and most linguistics after him) and we now freely speak of inner mental states, ideas, concepts, cognition, and meanings. Without a charity principle, Davidson has no way of guaranteeing that, absent social consensus, people mean the same thing when they say something. Charity, by ascribing the same true thoughts to all speakers, guarantees that there is intersubjective agreeement and therefore translation between languages^5^.

This is quite visible if we look at some of Davidson's statements on Charity. Charity, according to Davidson, is holistic, as can be seen from the following quotations: “we make sense of particular beliefs only as they cohere with other beliefs” (1980: p. 221); “the content of a propositional attitude derives from its place in the pattern”(Ibid.); “a belief is identified by its location in a pattern of beliefs; it is this pattern that determines the subject matter of the belief, what the belief is about” (1984: p. 169).

Furthermore, speakers are generally consistent:

crediting people with a large degree of consistency cannot be counted mere charity: it is unavoidable if we are to be in a position to accuse them meaningfully of error and some degree of irrationality (Davidson, 1980: p. 221)

Davidson notes that since we sometimes accuse people of being mistaken or of contradicting themselves, these charges can exist only against a background of consistency. Davidson allows for the presence of disagreement, error, and irrationality, [“it cannot be assumed that speakers never have false beliefs” (1984: p. 168) “of course a speaker can be wrong” (1984: p. 169)] but against the backdrop of general agreement, truth and rationality: “disagreement and agreement alike are intelligible only against a background of massive agreement” (1984: p. 137), “the methodological presumption of rationality does not make it impossible to attribute irrational thoughts and actions to an agent, but it does impose a burden on such attribution” (1984: p. 159).

Speakers are also rational, for Davidson, “successful interpretation [communication] necessarily invests the person interpreted with basic rationality” (Davidson, 2001: p. 211). Consistency, rationality, and coherence go hand in hand. Davidson remarks that “we necessarily impose conditions of coherence, rationality, and consistency” (Davidson, 1980: p. 231). In fact, the assumption of rationality, having beliefs, and intentional communication in an agent is founded by charity:

If we cannot find a way to interpret the utterances and other behavior of a creature as revealing a set of beliefs largely consistent and true by our own standards, we have no reason to count the creature as rational, as having beliefs, or as saying anything (Davidson, 1984: p. 137).

Because it needs to rely on observable behavior, Charity is based on public (social) assent:

A theory for interpreting the utterances of a single speaker, based on nothing but his[/her] attitudes toward sentences, would, we may be sure, have many equally eligible rivals, for differences in interpretation could be offset by appropriate differences in beliefs attributed. *Given a community of speakers with apparently the same linguistic repertoire, however, the theorist will strive for a single theory of interpretation* […] What makes a social theory of interpretation possible is that we can construct a plurality of private belief structures: belief is built to take up the slack between sentences held true by individuals and sentences true (or false) by public standards. […] Attributions of belief are as publicly verifiable as interpretations, being based on the same evidence: if we can understand what a person says, we can know what he[/she] believes (1984: p. 153; my emphasis, SA).

Davidson's argument here echoes Wittgenstein's against a private language. A language with one speaker could not be said to exist, because there would be no checking the meanings of the signs. Evnine (1991: pp. 105–108) acutely notes that Davidson, having acknowledged clearly the importance of the social aspect of language, goes on to more or less completely reject the idea of convention (Davidson, 1986), shifting his attention back from the social aspect of language to the individual role.

Charity forces the interpreter to attribute to the interpretee a set of true beliefs. Davidson seems to waver as to whether the truth of the speaker's beliefs is assumed relative to the interpreter's set of beliefs: he speaks of “assigning truth conditions to align sentences that make native speakers right when plausibly possible, according, of course, *to our own view of what is right*” (1984: p. 137; my emphasis SA) and of interpreting the behavior of another as “revealing a set of beliefs largely consistent and true *by our own standards*” (Ibid.; my emphasis, SA). Elsewhere, however, Davidson states that the speaker is objectively right: “massive error about the world is simply unintelligible” (1984: p. 201) and “successful communication proves the existence of a shared, and largely true, view of the world” (Ibid.) Davidson's position that Charity guarantees the truth of most beliefs of a community has not gone without challenges, e.g., McGinn (1999: pp. 178–179; 180–196). McGinn rejects the truth claim, but accepts the consistency and rationality claims in Davidson's account.

In my mind, the objections to the claim that the principle of charity requires speakers to attribute to each others true beliefs are misguided. Obviously Davidson never graded a set of midterms in my introduction to linguistics class, otherwise he would not have maintained that the possibility of “massive error” (Davidson, 1984: p. 197) is ruled out. However, this is beside the point. I understand Davidson as saying something much deeper and interesting than the obviously erroneous claim that people cannot be massively wrong about something. What I think Davidson meant (or should have meant, see below) is that even in order for my students to be massively wrong about linguistics they have to be massively right about way more things than linguistics in order to be counted as having the possibility to be right or wrong about linguistics. So, for example, they would have to have the true beliefs that linguistics exists, that the exam exists, that I exist, that exams are graded, that one wants to score well on an exam, etc. Here my interpretation of Davidson is along the lines of Wittgenstein's recognition of an unquestionable background of knowledge which anchors the very possibility of doubting something (e.g., Wittgenstein, 1969: p. 94; Stroll, 1994: p. 180 makes the connection to Davidson's charity).

Davidson himself seems, however, to deviate from this sort of reading, when he asks, rhetorically, “how clear are we that the ancients (some ancients) believed that the earth was flat? *This* earth? (…)” (1984: p. 168) and continues to argue that *our* earth is a planet in the solar system, etc. and if one does not have these beliefs, then (holistically) one cannot really be thinking about the same earth. But this line of reasoning is unnecessary: why not simply concede that some ancients were wrong about a few thousand beliefs and nonetheless right about millions of others (e.g., that the earth exists, that it is larger than one can walk in several days, etc.)?

Within Davidson's model, interpreters (speakers and hearers) need to attribute to one another beliefs that “minimize disagreement” (1984: p. xvii) or “maximize agreement” (1984: p. 101): “a good theory of interpretation maximizes agreement. Or, given that sentences are infinite in number (…) a better word might be optimize.” (1984: p. 169).

Finally, Davidson notes that his account of interpretation via charity goes against relativism (1984: p. 197); however, he also notes that this does not amount to a universalist view (198). Lukes (1982) considers the important implications of Davidson's Charity and Grandy's humanity against relativism (i.e., the possibility that there exist radically different cultures, logics, or worldview across which ideas are untranslatable). Rationality and some beliefs need to exist across the board (i.e., to be part of the definition of humanity), although obviously not all beliefs.

Finally, one may object to my treatment of charity principle proposed by philosophers as linguistic principles, but in fact it has been argued, convincingly to my mind, that this is precisely what Davidson's intention was (or at least that this is the outcome of his views):

[Davidson's] tacit equation of [the charity principle] with his own views about the constitutive role of rationality in determining what sentences we can be understood as holding true further blurs the nature of the Principle and *makes it seem more like a general maxim guiding interpretation* (Jackman, 2003; my emphasis, SA).

This concludes our discussion of the principle of charity, as proposed by Davidson. It might be interesting, however, to reflect briefly on specific linguistic aspects of charity. Let us recall that Davidson points out that charity forces us to attribute “coherence, rationality, and consistency” to the speakers we are engaging in conversation with as well as substantive knowledge about the world. The attribution of rationality entails the attribution of some sort of set of Gricean principles/maxims, since the principle of cooperation and/or the maxims are characteristics of rational communication. This is familiar ground, which I will not repeat in this context. The attribution of coherence is, in a sense, part of the injunction of speaking to the point (relevance) but in a different perspective it is responsible for the need to assume that a speaker is, say, answering a question even if prima facie the utterance does not seem to meet the requirements of coherence and/or relevance. In other words, the option of assuming that the speaker is incoherent is literally the dispreferred option. Finally, the assumption of consistency is perhaps the most intriguing in the linguistic aspects of charity. Essentially, it boils down to the assumption that the speaker is using language units in the same way, through the exchange, so that the meaning and/or reference of the units does not change during the exchange. In a more sophisticated sense, it also requires that we attribute to a speaker the meanings that we know that speaker to intend. A feature of (some) uncharitable readings is that they attribute to the speaker meanings that the speaker would not have meant. For example, the Wikipedia article on “Controversies about the word *niggardly*”^6^ reports several instances of speakers attributing racist intentions to those who used the word, apparently under the misconception that it would be etymologically related to a slur against African Americans^7^.

Charity and satisficing form the two bounding principles that constrain the expansive tendencies of relevance and abduction, seeking for features of the environment to make sense of the exchange or of the text. If we had not already introduced one metaphor, we could suggest thinking about the centrifugal (expansive) and centripetal (bounding) forces in physics that define the orbits of celestial bodies. In what follows we will examine a few examples of context definition with emphasis on the bounding, centripetal forces.

#### Examples of context derivation and bounding

Let us begin by a non-controversial example: deictics are by general agreement context-sensitive. Consider now the quantifier “everything” in the following examples (short hand indication of the “context” is given after the equal sign):

- Everything = “all that exists” (Wikipedia, consulted April 16, 2015)
- Make me one with everything = customer to hot-dog vendor
- I lost everything = investor after a stock market crash
- Everything = answer to the question: “Dr. Attardo, what will be on the test?”
- I know everything = husband to unfaithful wife.

Example (a) is Fetzer's un-delimited account in which the quantifier refers literally to every entity in the universe. In example (b) the transactional situation of purchasing provides a justification for the direct request, while the hot-dog vendor cart situation provides the antecedent for “one” (i.e., one hot-dog) which in turns provides the boundary of the expansion of everything to toppings by its affordances (see Attardo, 2005 for an analysis of an “everything” bagel. along the same lines). Note that in order for the reading to go off we must charitably not impute to the customer the reading in (a). Example (c) is different, in the sense that the situation in which the unlucky investor utters it is very salient, as presumably many speakers are discussing the market crash, newspapers and other media are commenting on it, etc. Again, relevance provides us with a direction to look for a referent for “everything”: the investor could have lost his/her suitcase, his/her laptop, or any set of things that form a group, but since we are talking about the stock market the most relevant reading is that the investor lost in his investments (and note how that shifts the meaning of “lost” from a literal sense of losing to a metaphorical one, since obviously it is not the case that the investor cannot find his/her stocks or municipal funds anymore, but that the value thereof is greatly diminished). Once more charity bids us to ignore the fact that prima facie (c) is likely to be false since it is unlikely that all of the investments lost all of their value. Generally, even bankrupt companies retain some assets that are worth a small fraction of the valuation of the company. Thus, (c) is to be taken as an exaggeration corresponding literally to “the value of my investments has dropped significantly and to the point that they are unlikely to fulfill the purposes for which I had invested these sums.”

Example (d) shows very clearly both the importance of charitable reading and of the affordances of the term “test.” Let us start by the fact that tests generally consist of questions about a subject. The situation bounds the test to an academic test in the humanities (as opposed to an endurance test in the marine corps, for example). The students are trying to determine what topics will be on the test. Abductively, we can infer that they are interested in this information in order to study those subjects (we will ignore if they will the neglect the other subjects, or if they merely wish to fine-tune their preparation). My response is cooperative (it provides them with the relevant information, it is clear, to the point, succinct, and truthful), if adversarial (I deny that there might be topics that should be prepared to the exclusion of others). However, note how the students must charitably attribute to me a bounded interpretation of “every topic covered in class” or “every topic on the syllabus” or “every topic in the book.” Had they failed to do so, they could have come to the conclusion that every topic in their major might be on the test (a relevant interpretation, but that would be irrational on my part, as such test are not ordinarily given to students in a regular class) or perhaps every topic in the discipline (again, a relevant interpretation, since the course was called “Introduction to linguistics” but again one that non-charitably would impute irrational or abnormal behavior on my part).

Finally, in example (e) we must imagine a situation, common from many narratives, in which a husband has discovered evidence of the unfaithfulness of his wife (obviously, the example also works with the genders reversed). Clearly the relevant, bounded interpretation is that he husband knows everything about the wife's affair. Note how, if the wife's response were, “Oh really? What's the square root of 1243?,” the assumption of relevance and boundedness would disappear. But let us now consider another response by the wife, who presumably has a PhD in logic, and might object, “No you don't. For example, you do not know how many times I have made love to Arthur, nor do you know what my pet name for him is!” The wife's objection is technically correct, since the topics she lists are within the domain of the relevant information. However, her response violates the maxim of charity, because it does not maximize the instances of shared true propositions between her and her husband. Indeed, by assuming that her husband means “I know everything that is important about the fact that you are having an affair” the number of shared true propositions would increase. Note how, once again, the important abductive inferential work done by charity to narrow down the domain of applicability of the statement^8^.

All these example highlight the dynamic, changing process whereby context is determined and bounded for the communicative purposes of and by the dynamics of the interaction of the speakers/participants. An observation should also be made about the partially facetious nature of some of the examples. Humor is a mode of communication that deliberately switches between one set of expectations (a reading of a text, for example) and another set of expectations that are different enough to be incongruous in relation to the first set. The switch can be achieved or merely partially explained or justified in a number of ways, for example through an ambiguous term, but also through a number of mechanisms, only some of which have been identified and described in the literature. Because of this switch in which the first interpretation of the text is rejected in favor of a second one, the context of the text also changes radically, although not entirely. Thus, in keeping with the metaphor introduced at the beginning of the essay, humorous switches between interpretations of texts may shed some indirect light on the processes that constitute context.

To illustrate this claim, and to provide a further example of the inferential processes in deriving context, we will examine a joke. The text is taken from a monolog delivered by a famous Italian comedian, who goes by the name of “Crozza,” on October 25th, 2014 on La7, an Italian private television channel. The entire clip of the performance is available online at http://www.la7.it/crozza/video/leopolda-renzi-come-steve-jobs-25-10-2014-139314.

The fragment we are interested in occurs within the times 1:13–1:45. Below is my translation of the relevant parts of the text.

Renzi said: we will begin the proceedings in a location that will remind us of a garage. A garage is a symbol of a place where ideas become startups, create employment. He [Renzi] thinks he is Steve Jobs. It's clear. (…) Perhaps, the comparison with Steve Jobs is appropriate. Since Renzi has been there, every year a new thinner model of the Democratic Party is released.

The performance occurs in a relatively impoverished context, with Crozza alone on an empty stage. During the first part of the text, relevant quotations from the speech of Italian Prime Minister Renzi are projected on the screen, behind Crozza. The quotes are from *Repubblica*, a prestigious daily newspaper. Obviously, the function of these quotes is to show that what Crozza is saying is true and that the PM did in fact say those things. Until the last sentence, Crozza is establishing a script (or frame) for political governance. Within this script, the creation of new and innovative employment opportunities figure prominently as positive actions that politicians may undertake to stimulate the economy and increase the well-being of the citizens. In turn, the context of “political discourse” is broadly activated. Then Crozza introduces the second script: Renzi thinks he is Steve Jobs. Since Steve Jobs is a great entrepreneur who created one of the most successful companies on earth, Renzi's comparison of himself with Jobs is inferred by the audience to be a case of megalomania and hence ridiculous. Note the shift from the political discourse context to the psychology of Renzi. After another jab at Renzi, which I have not included for simplicity, Crozza returns to the comparison between Renzi and Steve Jobs, introduced as a joke in the first part. Now Crozza says the comparison is actually accurate. In other words, Crozza is now claiming that there is at least one trait in which Renzi and Steve Jobs resemble each other. Since Jobs is high status individual, the comparison will elevate Renzi's own status. Crozza then reveals that the similarity lies in the parallelism that both Job's company, Apple, and Renzi's party, the Partito Democratico (PD), release a thinner model each year. Of course, while in technology thinner is better, in politics, thinner means less electors, which is of course bad. Note again how the introduction of the scripts for technological gadgets and political parties shifts the context, narrowing it down until it is pinpointed on the PD's loss of electoral share, since Renzi's appointment as Prime Minister. Note also, how charity not only bounds the limits of the context to the shrinking of the Prime Minister's Democratic Party, but also forces the hearer to accept the somewhat forced parallelism between Apple's releasing thinner telephones every year and the Democratic Party getting smaller every year. To deny the validity of the parallelism would be a serious violation of charity, as it would reduce Crozza's point to nonsense.

## Conclusion

Hopefully, to use the metaphor of non-foveal vision we have presented a set of principles and mechanisms that surround context and that operating in conjunction, determine it, if not completely, at least to a large extent. While this is not the same thing as describing context, it is a meaningful step in the direction of being able to determine what speakers do when they think-within-context.

Recapitulating briefly, speakers subconsciouly generate a mental construct of context using the concrete situation they are in, in al its richness, the semantics of the utterances, all inferences and abductive implicatures they can draw from those led primarily by the assumption of relevance, but by cooperation at large. The output of these, expansions is bounded by satisficing and by the need to provide charitable interpretations of the speakers' (linguistic or non) behavior. Generally speaking this mental construct is never above the threshold of consciousness, but some features in it can be brought to the speaker's attention through humor or other marked situations.

I have based this account of the concept of context on Davidsonian charity which I believe to be a significant addition to the related concepts of cooperation and rationality which are necessary to process implicatures and generally speaking the pragmatics of texts. However, care should be taken when applying a concept developed by philosophers within philosophy to a more empirical field such as linguistics. First, it is quite possible that my account of the principle of charity might not match exactly what Davidson says about it and/or might not dovetail exactly with other things Davidson said that are connected with it. As I have said elsewhere about Grice and my reading of his work, as it pertains to the principle of cooperation, sometimes it is necessary to read what Grice or Davidson *should* have said. Reading a work should be grounded in a reading as precise and close to the intentions of the author's as possible, but that should not stop one from deviating from what the author says, if it is possible to improve on it. Needless to say, one should be clear, as I hope I have been, when one is doing which. Second, the use I have made of the principle is probably not one of the uses that Davidson or more generally philosophers would have intended. About this, I am unapologetic. Using a door as a table may be a good or a bad idea, but its success depends on how well it functions as a table, not on whether its door-related teleology has been fulfilled. Third, by using a concept from one theory of philosophy, one does not enter in a binding contract requiring him/her to solve all the problems related to that theory. If the door sticks, as a table user, I am not morally, ethically, or otherwise bound to fix the sticking problem (my advice: lightly plane the offending surface). Fourth, using a concept from a theory does not require one to adhere to the rest of the theory: if I am using a door as a table, I have not committed myself to purchasing the house the door comes from. Specifically and outside of metaphor, I do not buy the behaviorist undercurrent in Davidsonian semantics.

## Author contributions

The author confirms being the sole contributor of this work and approved it for publication.

### Conflict of interest statement

The author declares that the research was conducted in the absence of any commercial or financial relationships that could be construed as a potential conflict of interest. The reviewer, MT and handling Editor declared their shared affiliation, and the handling Editor states that the process nevertheless met the standards of a fair and objective review.

## Footnotes

^1^The metaphor is used also in Schegloff (1992: p. 223).

^2^Schegloff (1992; 1997) is concerned primarily with the difference between the view of context for the purpose of analysis (the analysts' view) as opposed to the view of context as integral to the unfolding of the speech event (the participants' view). Moreover his approach belongs to a tradition that does not fit easily within the methodological underpinning of contemporary linguistics and in many ways is antagonistic to (some of) them. It would be far too time consuming to review these in any detail, not to mention that none of the discussion would be particularly relevant to the present article.

^3^The sentence occurs on p. 65 of the Beadle's monthly, volume 3, which appeared in 1867, in New York. The author is Kate Putnam Osgood. *Our New House, A Story*.

^4^Interestingly, this presents a serious problem for Schegloff's (1992: p. 215) demand that “demonstrable relevance to the participants” be the warrant for claims about context. Ideology can be very relevant to the establishment of context, but by definition, it will be invisible to its followers.

^5^One might ask, since I assume that mental states exist, why use charity at all? The answer is that we need charity, but for different purposes than Davidson did.

^6^Wikipedia. *Controversies About the Word “Niggardly”*. Accessed December 26, 2015.

^7^Interestingly, the Wikipedia article also reports that some would be using the term as a “code” word for the slur, essentially to provide deniability. In that case, the reading that attributes racist intentions would not be uncharitable. Incidentally, uncharitable readings are not inherently bad. They may serve political purposes. For example, the Democrats made much of then-vice-President Dan Quayle's misspelling of the word “potato” in a 1992 school photo-op. Obviously and charitably, Quayle knew the correct spelling, but accusing him of ignorance served the Democrats' agenda.

^8^One may object that since the wife has deceived the husband, there is no need or point to be charitable. This objection misses the point entirely. Charity is needed to communicate, not to be nice to people.
